# Japan Energy Database with hourly and municipal-scale estimates to support local energy system analysis

**DOI:** 10.1038/s41597-025-06294-w

**Published:** 2025-12-02

**Authors:** Ryoga Ono, Hiroaki Onodera, Koyo Kikuchi, Yudai Morita, Rémi Delage, Toshihiko Nakata

**Affiliations:** 1 Department of Management Science and Technology, Graduate School of Engineering, Tohoku University, 6-6-11-815 Aramaki Aza Aoba, Aoba-ku, Sendai, Miyagi 980-8579 Japan; 2 Social Systems Division, National Institute for Environmental Studies, 16-2 Onogawa, Tsukuba, Ibaraki 305-8506 Japan; 3 Department of Integrated Engineering Science, Graduate School of Engineering Science, Akita University, 1-1 Tegata Gakuen-machi, Akita-shi, Akita, 010-8502 Japan

**Keywords:** Energy supply and demand, Energy management

## Abstract

Achieving “carbon neutrality” in Japan is complicated by fragmented institutional responsibilities and the absence of municipal‐scale energy data. Decarbonization is led by the Ministry of the Environment, reflecting a segmented administrative framework. Moreover, sectoral energy consumption data are not officially published at the municipal level, hindering evidence-based local policy development. To overcome these challenges, we have developed the Japan Energy Database, a comprehensive dataset of energy supply and demand estimates for all 1,741 municipalities. This open-access dataset enables: (1) locally grounded energy strategies and implementation, (2) assessment of regional renewable energy potential and utilization, and (3) evidence based support for resilient, sustainable social transitions. It integrates national statistics, spatial information, and engineering assumptions to estimate final energy consumption in transportation, industrial, residential, and commercial sectors. We detail the data-construction methodology and validate its internal consistency and policy relevance. The dataset provides a foundation for decentralized, data-driven energy planning and region-specific decarbonization pathways in Japan.

## Background & Summary

The design of energy systems, their social implementation, and the pursuit of “carbon neutrality” have become critical challenges. In Japan, the Ministry of Economy, Trade and Industry (METI) oversees energy utilities, while the Ministry of the Environment (MOE) is responsible for “decarbonization”. Moreover, METI focuses on national-scale initiatives, while MOE extends its scope to local municipalities. A major challenge is the lack of energy supply and demand data at the municipal level, making it difficult to achieve decarbonization in local communities because the energy consumption data that contributes to carbon emissions remain unclear. The municipal emissions inventory^1^ published by the MOE is calculated by allocating the national total emissions based on the activities of local prefectures, targeting only CO₂ emissions. While these are useful to some extent, they lack any underlying energy supply and demand data in principle. As a result, while there is a strong emphasis on awareness campaigns promoting energy saving and eco-driving, there is no scientific method to formulate regional energy strategies or decarbonization strategies, or to verify their effectiveness. This has left enthusiastic consumers and local leaders feeling confused. Furthermore, in rural areas, conflicts are increasing between local residents opposed to the installation of large-scale solar, wind power plants and other renewable power generation facilities. This can be attributed to dissatisfaction stemming from a lack of understanding of the value and benefits of renewable energy, which are not shared within local communities. Since the Great East Japan Earthquake in 2011, there has been a surge in interest in introducing autonomous, decentralized energy systems to enhance the resilience of local communities, based on the experience of nationwide, long-term major disruption in energy supply

To address this issue, we have developed the Japan Energy Database (JED)^2^, Japan’s first regional energy supply and demand dataset targeting all 1,741 municipalities. Notably, the data sources used in this dataset are based on officially published statistics, ensuring transparency and reliability. A linked visualization tool automatically generates energy flow diagrams based on the dataset, enabling even non-specialists to intuitively grasp local energy supply and demand characteristics. The following three effects are expected from this dataset: 1. Support for the formulation of practical and effective regional energy strategies. The dataset provides knowledge and evidence-based information for discussions among stakeholders, including national and local governments as well as business operators. 2. Quantification of the value of renewable energy resources available in the region. 3. Realization of a paradigm shift in social structures that enhances the resilience of regional communities and promotes carbon neutrality, while ensuring economic rationality through energy system design.

## Methods

The following section describes the methods used to estimate renewable energy potential and energy consumption at the 1,741 municipal level. In this study, we have used the standard terminology of “renewable energy” following the definition by the International Energy Agency (IEA), which includes solar, wind, hydro, geothermal, and biomass. In addition, the hourly energy data for each municipality is also presented in 2019. Estimation of energy consumption and installed capacity of renewable energy was carried out for the years 2013, 2016, and 2019. While the datasets differed over the years, a consistent methodology has been applied throughout.

### Renewable energy data

The JED includes renewable energy technical potential and installed capacity for solar photovoltaics (PV), onshore wind, offshore wind, geothermal energy, run-of-river hydropower, municipal solid waste (MSW), and woody biomass at the municipal level. Technical potential data for solar PV, onshore wind, geothermal, and run-of-river hydropower were provided from the Renewable Energy Potential System (REPOS)^3^ operated by the MOE. Offshore wind potential was calculated following the methodology of REPOS and spatially allocated to municipalities using Voronoi splitting. MSW potential was estimated based on the amount of combustible waste delivered to public waste treatment plants^4^. Woody biomass potential was based on the annual forest growth estimated in our previous study^5^. The renewable energy capacity installed each year was sourced from the Feed-in Tariff (FIT) certified capacity records^6^. For onshore wind, the capacity data of non-FIT-certified plants were provided by the Japan Wind Power Association (JWPA). In estimating annual electricity generation, the natural capacity factor was assumed based on the values provided by the Japan’s Agency for Natural Resources and Energy^7^. The MSW is an exception, for which the installed capacity and electricity generation were obtained from official statistics^4^.

Hourly natural capacity factors for each variable renewable energy source were derived from 30-minute meteorological satellite data^8^. The input data includes solar radiation (global, direct, and diffuse components) and wind speed. The spatial resolution is 1 km^2^, and the mean value within each region is used as the representative estimate.

#### Solar photovoltaics

The natural capacity factor of photovoltaic power generation was estimated at the municipal level based on Bett and Thornton^9^. In this reference, the calculation considers four components: the rated module efficiency under standard testing conditions (STC: irradiance of 1000 W/m², cell temperature of 25 °C, and AM1.5 spectrum), the efficiency of other connected equipment, the relative efficiency, and the total solar irradiance. The efficiency of other connected equipment was assumed to be 0.9.

The relative efficiency depends on the panel surface temperature. The panel surface temperature was derived using a thermal energy balance approach, incorporating ambient air temperature, ground temperature, and sky temperature. Heat convection was modeled by considering both natural and forced convection. For the latter, wind speed at a height of 1 m was estimated from the standard 10 m wind speed^8^ using the Hellmann equation. Since wind speed depends on land use through surface roughness, the exponent was derived from raster data^10^ representing local surface conditions.

Total solar irradiance was calculated as the sum of direct, reflected, and diffuse components on an inclined surface. Direct solar irradiance was calculated assuming a panel tilt angle of 30 degrees across all locations. Reflected irradiance was obtained by applying an albedo coefficient of 0.2, uniformly assumed for all locations, to the total ground-level irradiance. Diffuse irradiance was estimated by accounting for isotropic sky radiation, circumsolar brightness, and horizon brightening. The methodology for calculating the diffuse irradiance follows the model developed by Perez *et al*.^11^.

#### Onshore and offshore wind power

The relationship between wind speed and power generation is expressed through the power curve, which incorporates turbine characteristics such as rotor swept area. The estimation method follows previous research developed by Delage *et al*.^12^, where the cut-out wind speed for both onshore and offshore turbines is set at 25 m/s.

Onshore wind power was modeled using a representative wind turbine with a rated capacity of 4.2 MW (IEC Class IIA), while offshore wind power was based on a 10 MW turbine (IEC Class IB). The hub heights were set at 90 m for onshore and 140 m for offshore turbines.

For offshore wind, the target marine areas were selected according to criteria set by the MOE^13^. These included locations with wind speeds exceeding 6.5 m/s, within 30 km of the coastline, sea depths less than 200 m, and areas outside national and quasi-national parks. It should also be noted that the potential estimation does not account for the presence of isolated grids on certain islands.

#### Run-of-river hydropower

The time-series distribution of run-of-river hydropower was estimated based on river flow volumes within each municipality and its neighboring regions. Flow data were originally collected from 2,286 measurement points, but only 670 points with complete year-round data for 2019 were used in the analysis^14^. The rated flow rate at each point was set to the annual average flow rate.

### Energy consumption data

**Fig. 1:**
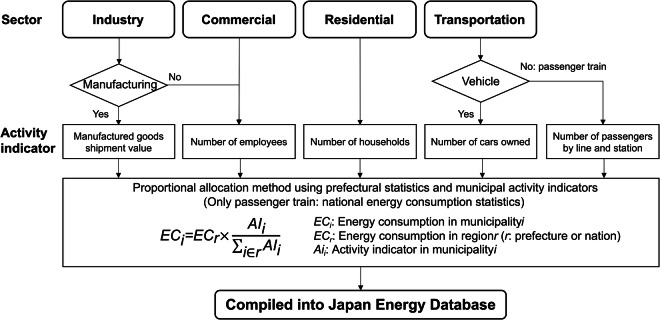
The process of estimating municipal energy consumption by proportional allocation method.

Energy consumption data, excluding aviation, were estimated using a proportional allocation method based on prefectural^15,16^ or national statistics^17^ and municipality-specific activity indicators. Aviation-related energy consumption was allocated from individual airports^18^ to municipalities. For the industrial sector, the manufacturing industry was represented by the value of manufactured goods shipments^19^, while the non-manufacturing industry and the commercial sector were represented by the number of employees^19,20^. In the residential sector, the number of households^21^ was used as the activity indicator. In the transportation sector, passenger train activity was represented by passenger counts by line and station^22^, and all other transport activity was represented by the number of owned cars^23,24^. The process of estimating energy consumption is illustrated in Fig. 1. The data covers four sectors – transportation, industrial, residential, and commercial – and each sector is further divided by energy source: electricity, liquefied natural gas (LNG), coal, and crude oil. Additionally, the industrial and commercial sectors are subdivided into 29 categories, while the transportation sector is divided into 5 categories. This approach has been adopted by Onodera *et al*.^25^.

The composition of electricity production was obtained from publicly available data for Transmission System Operator (TSO) regions, which are based on Japan’s former General Electric Utilities. Residential energy consumption was further broken into heating, cooling, hot water supply, and others, according to regional breakdown ratios defined for nine regions^26^.

Hourly electricity consumption for each municipality was derived from the consumption profile of the corresponding TSO region. Consequently, all municipalities within the same TSO region shared an identical time-series distribution pattern.

## Data Records

The dataset is available in the Japan Energy Database repository^2^. It includes data for 1,741 municipalities, as well as aggregated data at the prefectural, TSO, and national levels for the years 2013, 2016, and 2019, together with an hourly dataset for 2019. The datasets for 2013, 2016, and 2019 contain prefectural and municipal names and codes, renewable energy potentials and installed capacities, and energy consumption disaggregated into four sectors and 34 categories, as well as four fuel types: electricity, liquefied natural gas, coal, and crude oil. The 2019 hourly dataset is structured with municipal codes as columns and hourly values as indices. The “JapanEnergyDatabase” folders provide the 2013, 2016, and 2019 datasets organized by region, while the “Time-series” folders include hourly datasets for offshore wind, onshore wind, solar photovoltaic, run-of-river hydropower, and electricity demand across all 1,741 municipalities.

## Technical Validation

In this section, the methodology of the JED, which estimates municipal-level energy consumption by downscaling from the prefectural or national level, was validated to ensure its applicability and consistency. Their validation focused on four end use sectors: transportation, industrial, commercial, and residential sectors.

Although the methodology of the JED - excluding aviation fuel - is based on the simple assumption of a linear relationship between sectoral energy consumption and a single activity indicator with the y-intercept set to zero, actual energy consumption is affected by multiple factors, which may lead to discrepancies between actual and estimated values.

To validate the method applied in the JED, we examined the correlation between measured energy consumption and activity indicators using available statistical data. Since no official data on sectoral energy consumption at the municipal level are publicly available in Japan, we used alternative sources: Eurostat data for the transportation^27,28^ and industrial sectors^29,30^, and Danish data from the Energi Data Service and Statistics Denmark for the commercial^31,32^ and residential sectors^31,33^. Because the JED provides detailed energy consumption data for 1,741 municipalities, particularly in the industrial and transportation sectors, validating it at the municipal level is challenging. Therefore, Eurostat was used to supply aggregated national-level data from 27 European countries with differing energy consumption scales and types. Additionally, Danish data were considered appropriate for validation, given that average annual temperature (Denmark: 10.16 °C, Japan: 13.78 °C)^34^, primary energy consumption per person (Denmark: 37,286 kWh, Japan: 41,086 kWh)^35^ and average municipal population (Denmark: 59,200 people, Japan: 72,500 people)^36,37^ are comparable. However, of the 98 Danish municipalities, the four largest with exceptionally high populations (e.g., Copenhagen) were excluded from the analysis as outliers.

### Transportation sector

**Fig. 2:**
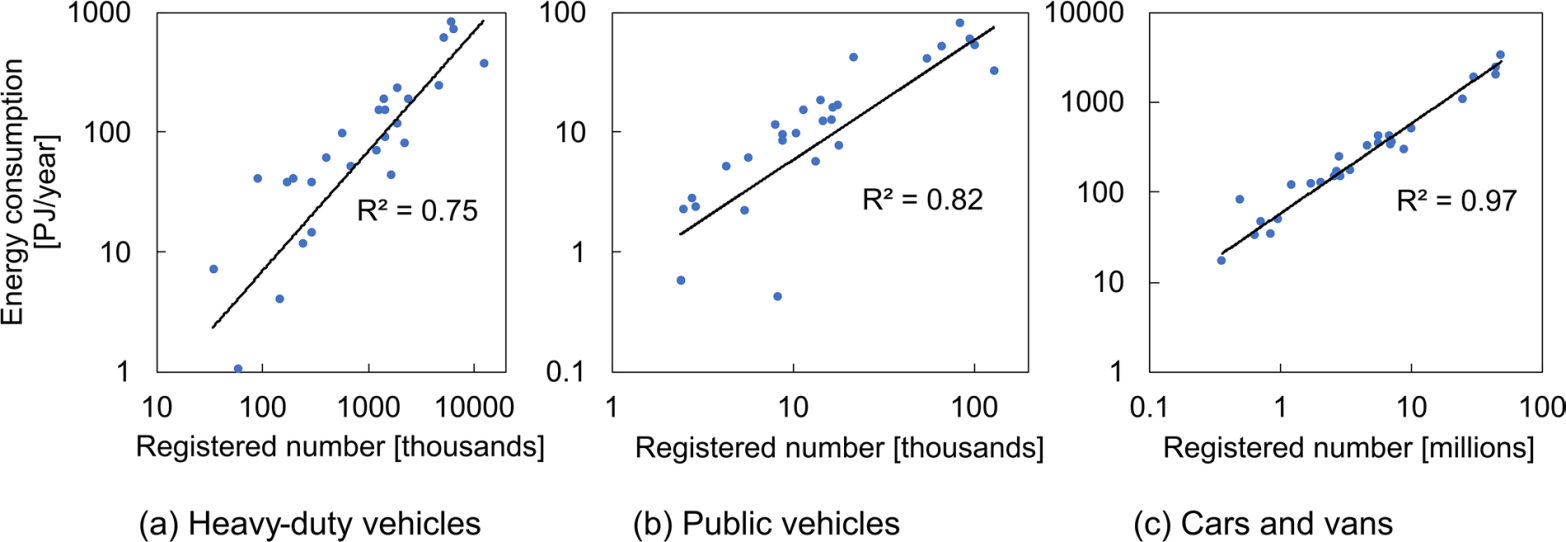
Energy consumption by vehicle type in the transportation sector. (**a**) Heavy-duty vehicles, (**b**) Public vehicles, and (**c**) Passenger cars and vans.

To evaluate the assumption of proportionality in the transportation sector, data on *cars and vans*, *heavy-duty vehicles*, and *public vehicles* were analyzed across various European countries. The relationship between fuel consumption and the number of vehicles of each type is illustrated in Fig. 2. The results indicated that the correlation coefficient for the cars and vans category was 0.97, showing a strong relationship with total energy consumption in road transportation and supporting the use of an activity indicator. Additionally, the correlation coefficients for heavy-duty vehicles and public vehicles were 0.75 and 0.82, respectively. Notably, 17 out of 27 countries exhibited deviations of less than ±20% from the regression line for total vehicles, suggesting that the proportional estimation method may entail an approximate error margin of 20%. Although the JED distinguishes between freight and passenger transport at the municipal level, this analysis could not differentiate small freight vehicles under 3.5 tons from passenger cars in the energy consumption data. Nevertheless, a significant relationship between fuel consumption and the number of vehicles was confirmed, suggesting that a similar correlation is likely to hold even when freight and passenger transport are distinguished.

### Industrial sector

**Fig. 3:**
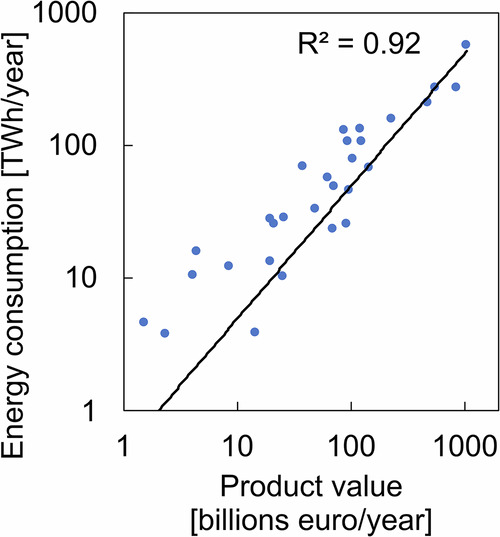
Energy consumption and composition in the industrial sector.

In the industrial sector, the shipment value of manufactured goods was used as the activity indicator. Figure 3 presents a cross-national regression analysis between total industrial production values and energy consumption, based on Eurostat data from European countries. The analysis reveals a high degree of proportionality, with a strong correlation coefficient of 0.92, suggesting that production value is a generally reliable proxy for estimating energy use at the municipal level.

### Commercial and residential sectors

**Fig. 4:**
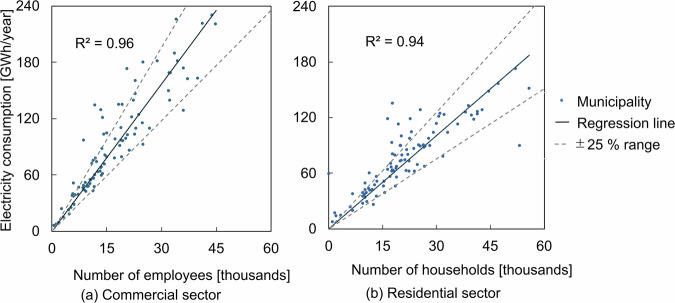
Simple linear regression analysis between electricity consumption and activity indicator of 94 municipalities in Denmark in 2019. Activity indicator as independent variable, electricity consumption as dependent variable and y-intercept set to zero.

Figure 4 shows the results of simple regression analysis of electricity consumption based on activity indicators in commercial and residential sectors. From the results, with high coefficients of determination of 0.96 and 0.94 in the commercial and residential sectors, respectively, indicated a strong correlation between measured energy consumption and activity indicators. Furthermore, 76% of municipalities in the commercial sector and 70% in the residential sector had estimated electricity consumption values that were within ±25% of the corresponding values predicted by the regression model. Among these municipalities, those with a small average building area per capita tend to be overestimated, while those with a large average building area per capita tend to be underestimated.

Based on these results, we considered that the methodology of the JED is valid not only in Denmark but also in Japan, where municipalities have a similar population scale. A limitation of this methodology is that regional characteristics, such as differences in building size and energy efficiency, may affect estimation accuracy. In particular, energy consumption per household in urban and rural areas may vary considerably due to differences in dwelling types, such as apartments and detached houses. Moreover, when municipalities with disproportionately large activity indicators are present within a prefecture, the influence of urban form and spatial concentration may not be adequately captured in the residential and commercial sector data.

## Usage Notes

On the JED web application (https://energy-sustainability.jp/)^38^, municipal-level energy balance sheets and hourly datasets are available for download, and some of the data are also provided with visualization features.

All datasets redistributed as part of the JED are openly available and compliant with their respective licenses. Some proprietary input datasets were used during processing, but these are not redistributed here.

Users should be aware that spatial heterogeneities such as urban form, vehicle stock, travel behavior, and building stock composition are not explicitly captured in the current dataset, and the estimates should be interpreted as representative values rather than bounded ranges. Uncertainty quantification, such as confidence intervals or upper and lower bounds, is not included in this version of the database. Nevertheless, clustering municipalities based on these characteristics and incorporating approaches from uncertainty analysis represent promising avenues for future refinement of the dataset.

## Data Availability

The datasets generated and analyzed in this study are publicly available on Zenodo (10.5281/zenodo.17746690)^2^.

## Code availability

Excel for Microsoft 365 version 2402, ArcGIS Pro version 3.3, and Python version 3.11 were used to develop the dataset, including geospatial information and analysis for technical validation. All procedures used to generate the dataset are fully described in the Methods section. As no custom code was developed, and only standard software operations were applied, no separate code files are necessary to ensure reproducibility.
